# Graphite flake self-retraction response based on potential seeking

**DOI:** 10.1186/1556-276X-7-185

**Published:** 2012-03-09

**Authors:** Tuck Wah Ng, Chun Yat Lau, Esteban Bernados-Chamagne, Jefferson Zhe Liu, John Sheridan, Ne Tan

**Affiliations:** 1 Laboratory for Optics, Acoustics, & Mechanics, Monash University, Clayton, VIC3800, Australia; 2 Department of Mechanical & Aerospace Engineering, Monash University, Clayton, VIC3800, Australia

## Abstract

The high elastic modulus and interlayer strengths of graphite flakes make them a durable solid superlubricant. Apart from this, they have configurable electrical properties, exhibit quantum Hall effects, and possess a myriad of useful photonic properties. The self-retraction behavior of graphite flakes can have significant impact on the creation of ordered stacks for various applications because any accidental or intentional displacement of the top flake over the stacks below may result in a misalignment of the carbon-carbon atomic arrangement which, in turn, can have influence over the electrical and photonic properties. It has also been revealed that there was a tendency of the displaced microflake to fail at times to return to its original starting position and orientation. Here, we elucidate this behavior by considering the influence of the interlayer potential forces based on minimal potential energy seeking. The maps of the parameters interrogated here provide the ability for precautions to be undertaken. They also potentially permit the creation of an array of microflake stacks in which the metastable states permit different information to be encoded by virtue of the differentiated photonic or electrical characteristics readable from each array site.

## Introduction

The many favorable mechanical, electrical, thermal, and biocompatible properties of graphite render it as an important material. Much work has been expended to investigate the superlubricity [1,2] between graphite layers where the high elastic moduli and interlayer strengths make it a durable solid lubricant. It also has configurable electrical properties [3-5], exhibits quantum Hall effects [6], and possesses a myriad of useful photonic properties [7-10]. Digressing to the mechanical property aspect of a closely related structure, the ability of nested shells in individual multi-walled carbon nanotubes (MWCNTs) to slide has been known for some time [11,12]. These MWCNTs have comparable superlubricity as graphite, in which the interwall shear strength against sliding ranges from 0.08 to 0.3 MPa. An interesting behavior of these extracted inner shells is in their ability to self-retract into the outer shells when the extracting force is removed [11]. This has been attributed to the action of van der Waals (vdW) interaction forces [11,12]. Such a feature has led to the conception of MWCNT-based actuator bearings [13] as well as nanoelectromechanical oscillators which offer the possibility of operating at frequencies in the gigahertz range [14,15]. More recently, a similar self-retraction capability was demonstrated on two graphite microflakes [16] of equal sizes. Although not mentioned previously, such a behavior can have significant impact in the creation of ordered graphene stacks for various applications. This is because any inadvertent or intentional displacement of the top flake over the stacks below may result in a misalignment of the carbon-carbon atomic arrangement which, in turn, can have influence over the electrical and photonic properties. It has also been revealed that there was a tendency of the displaced microflake to fail at times to return to its original starting position and orientation [16] unlike the MWCNT. Here, we attempt to elucidate this behavior by considering the influence of the interlayer potential forces.

## Method

**Figure 1:**
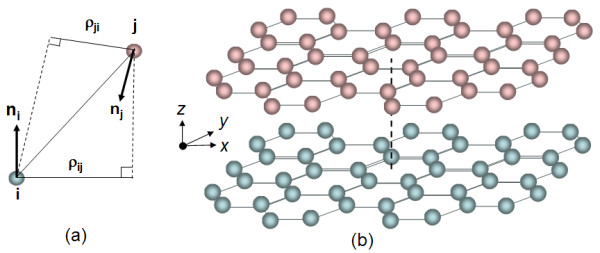
**The schematic description of the van der Waals interaction between the i-th and j-th atom**. Schematic description of (a) the van der Waals interaction between the i-th and j-th atom in the registry-dependent potential approach. A depiction of (b) a top graphite flake (pink) that is moveable relative to a bottom (blue) graphite flake.

**Figure 2:**
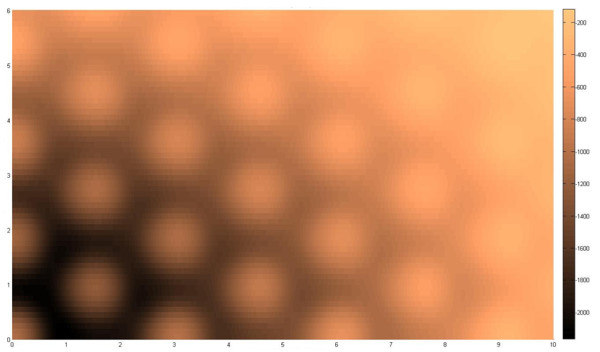
**The potential distribution (units in meV) when a top graphite flake is moved**. The potential distribution (units in meV) when a top graphite flake is moved relative to the bottom graphite flake of the same size (11*a *versus 6*a *in the × and y axis, respectively, where *a *= 0.142 nm) in the xy plane. The movement values are in terms of *a*. As movement should tend towards highest negative potential, the general landscape indicates a general tendency for the top flake to retract. However, the undulations imbue a possibility for it not to return to the exact original position and orientation.

The interlayer interaction between graphite layers has been investigated on perfect sheets [17-19] and on sheets with defects [20]. One method to model the interlayer interaction between graphite layers is to use a Leonard-Jones type of potential. However, such a model may not be sensitive enough to account for the registry of the honeycomb structures and its layers. It is generally accepted that the interlayer interactions are dominated by long-ranged vdW interactions. A registry dependent interlayer interaction potential can be parameterized (see Figure 1a) for layered carbon structures using [21,22]

(1) $$\begin{array}{c} V\left(r_{ij},n_{i},n_{j}\right)=e^{-\lambda \left(r_{ij}-z_{0}\right)}\left[C+f\left(\rho_{ij}\right)+f\left(\rho_{ji}\right)\right]-A{\left(\frac{r_{ij}}{z_{0}}\right)}^{-6}\\ \;\;\;\;f\left(\rho \right)=e^{-{\left(\rho /\delta \right)}^{2}\sum C_{2n}{\left(\rho /\delta \right)}^{2n}}\\ \;\;\;\;\;\;\;\rho_{ij^{2}}=r_{ij^{2}}-{\left(n_{i}r_{ij}\right)}^{2} \end{array}$$

where *V *is registry dependent interlayer potential, subscript *i *indicates any atom on bottom flake, subscript *j *indicates any atom on top flake, *r_ij _*is distance between atom *i *and atom *j, n *is normal vector of corresponding flake, *ρ_ij _*is transverse distance between two atoms, *f *is a function to estimate rapid decay of interlayer potential energy, *z*_0 _= 3.34 Å is equilibrium interlayer spacing, *λ *= 3.629 Å^-1^, *C *= 3.03, *A *= 10.238, *δ *= 0.578, *C*_0 _= 15.71, *C*_2 _= 12.29, and *C*_4 _= 4.933. The inter-atomic distance of carbon, *a *= 142 nm, provides a convenient depiction of geometrical size. In our simulations we apply a flake size of 11*a *and 6*a *in the × and y axis, respectively (see Figure 1b). Figure 2 traces the potential variations as one perfect graphite layer over one of the same size as a function of the sliding distances in the xy-plane. It can be seen that there is a general increase in potential (more positive indicating less attraction) with sliding distance, thus, accounting for the tendency of the moving flake to self-retract when the motive force to slide is removed. However, the profile contains local potential peaks and troughs. Hence, while the motive force is strong enough to surmount these valleys in the process of moving the flakes apart, the retraction process may engender the flake ceasing to move at the first local trough encountered in the return trip. If we assume the mass of the flake to be small, it is possible to depict the retraction process as a series of quasi-static steps. In each of these steps, which should be small enough, the nearby landscape is interrogated in six ways *δ*x = *δ*y = ± 0.01 Å, *δφ *= ± 0.5° to yield six potentials. In order to arrive at these values, we selected a few displaced positions and orientations and reduced the values until no changes in the return path. The flake can then be taken to advance to the incremental position or orientation based on the lowest of these potentials found if its value is lower than the current potential. Otherwise, the flake is assumed to have arrived at its final retracted state.

**Figure 3:**
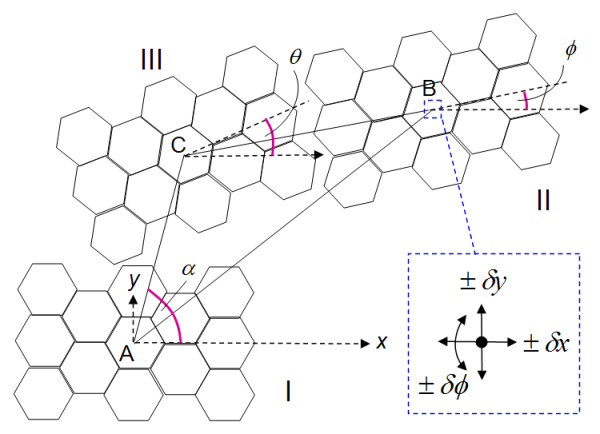
**Depiction of the top flake orignally at position I that is moved to position II**. Depiction of the top flake originally at position I that is moved to position II some x-y distance away and rotated by an angle *φ *to the *x*-axis. The flake is then allowed to perform a series of potential searches at the vicinity of the center of the flake about ± *δ*x, ± *δ*y, and ± *δφ *for sequential next position and orientation retraction that ends a rest position III. The parameters tracked are the center-to-center distance between positions I and III (AC), the angular displacement of the center-to-center positions of I and III (*α*), the center-to-center displacement between II and III (B,C), and the angular displacement at position III relative to the horizontal (θ).

In order to characterize the movement more systematically, we adopt the following approach (see Figure 3 in which the distances apart are exaggerated for clarity). The top flake is originally at position I. It is then moved to position II some x-y distance away and rotated by an angle *φ *to the *x*-axis. The flake is allowed to perform a series of potential searches at the vicinity of the center of the flake about ± *δ*x, ± *δ*y, and *δφ *to seek for the next position and orientation to retract to. This proceeds sequentially until the flake moves to a rest position III.

## Results and discussion

**Figure 4:**
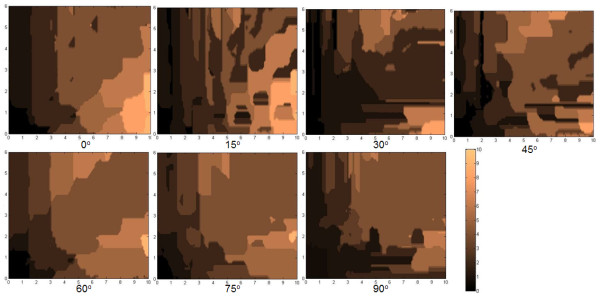
**The map of the center-to-center distance between positions I and III**. Map of the center-to-center distance between positions I and III (A,C) in Figure 3 at x-y distance away and rotated by an angle *φ *to the *x*-axis. The inability to self-retract to the original starting position is evident.

**Figure 5:**
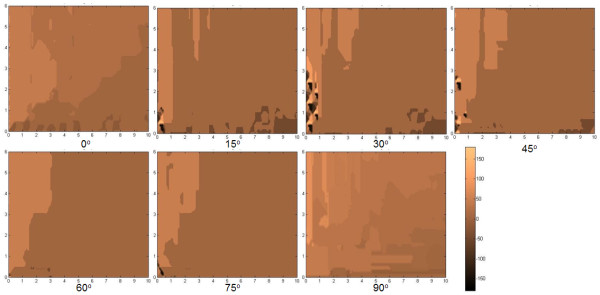
**The map of the angular displacement of the center-to-center positions of I and III**. Map of the angular displacement of the center-to-center positions of I and III (*α*) in Figure 3 at x-y distance away and rotated by an angle *φ *to the *x*-axis.

**Figure 6:**
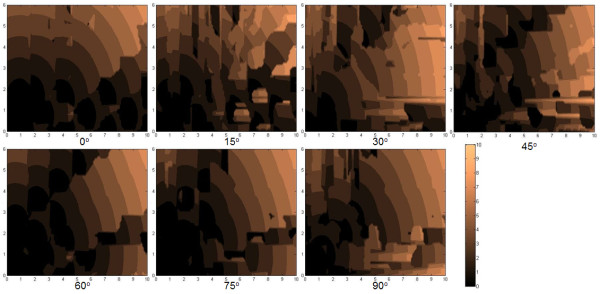
**The map of the center-to-center displacement between II and III**. Map of the center-to-center displacement between II and III (B,C) in Figure 3 at x-y distance away and rotated by an angle *φ *to the *x*-axis.

The parameters tracked are (a) the center-to-center distance between positions I and III (AC in Figure 3, (b) the angular displacement of the center-to-center positions of I and III (*α *in Figure 4), (c) the center-to-center displacement between II and III (BC in Figure 5), and (d) the angular displacement at position III relative to the horizontal (θ in Figure 6). These parameters were traced in relation to × and y distance as well as *φ *introduced. Only one quadrant of possible movement was explored due to symmetry.

In the plot of distributions of AC using *φ *= 0° in Figure 4, we find that there is an ability for the top flake center to self-retract to a high degree back to its original position center (e.g. <*a*). There is a somewhat reducing ability to do this when × and y are increased. This is logical as there is a greater opportunity for the flake to encounter more local minima during retraction even though the overall potential difference is higher with greater displacement. Nevertheless, one finds an easier capability to self -retract fully when displaced in the y as opposed to the × direction. This demeanor is rather preserved despite the angle *φ *changing, as seen in the other plots with values ranging from 15° to 90° at intervals of 15^°^. Overall, however, one finds that there are a myriad of states with which the top flake is able to stop at, depending on the displacing positions and orientations. The plot of distributions of *α *with *φ *in Figure 5 essentially tells of the quadrant the flake returns to. There were cases wherein if AC was small, the flake may move into other quadrants. Nevertheless, we did not find unexpected cases in which the flake could move to another quadrant when AC was large.

**Figure 7:**
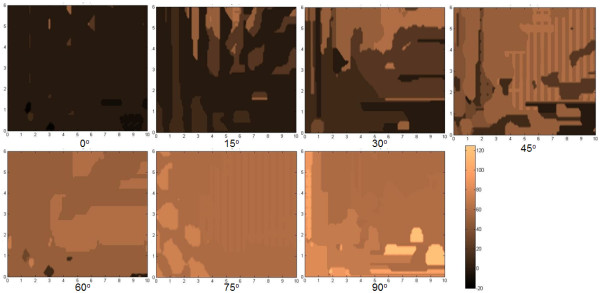
**The map of the angular displacement at position III relative to the horizontal (θ)**. Map of the angular displacement at position III relative to the horizontal (θ) in Figure 3 at x-y distance away and rotated by an angle *φ *to the *x*-axis.

From the plot of distributions of BC with *φ *= 0° in Figure 6, we find general rings which typically indicate that a flake that is pulled further apart will travel further to try to restore its original position. This is consistent with the potential map shown in Figure 2. Nevertheless one finds that there are islands when the flake is pulled more in the x-direction where BC is zero. This trend mirrors the finding given in Figure 4. From the plot of distributions of θ with *φ *= 0° in Figure 7, we find that there will generally be large islands of non-rotated states (i.e. θ θ 0°). The other plots indicate that the top flake to mostly θ to mostly rest at the angles with which φ is used. This result infers that the retraction process overall appears to seldom find *δφ *as the next lower potential step to advance to. However, the map also indicates that there are exceptions to this. We suspect that such cases should closely correspond when the top flake encounters a peak in its path and, thus, undergoes rotation more in order to find the next potential minima.

**Figure 8:**
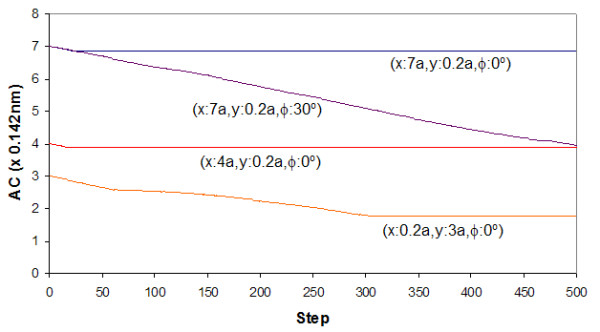
**The trace of AC**. Trace of AC in relation to step trajectories when the top flake was released at selected x-y distances away from the initial position and rotated by an angle *φ *to the *x*-axis.

**Figure 9:**
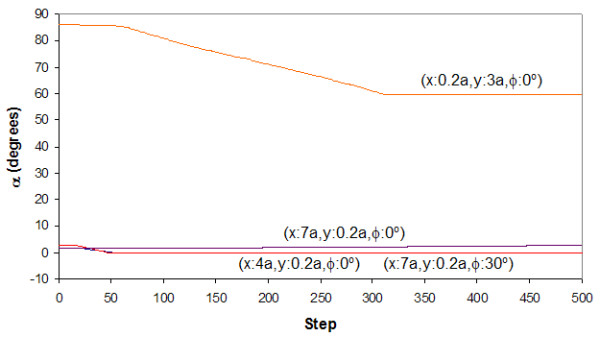
**The trace of *α***. Trace of *α *in relation to step trajectories when the top flake was released at selected x-y distances away from the initial position and rotated by an angle *φ *to the *x*-axis.

**Figure 10:**
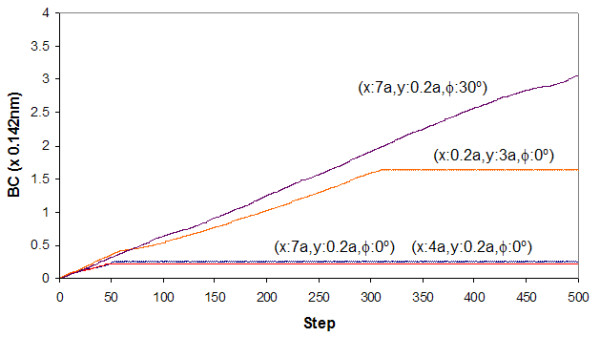
**The trace of BC**. Trace of BC in relation to step trajectories when the top flake was released at selected x-y distances away from the initial position and rotated by an angle *φ *to the *x*-axis.

**Figure 11:**
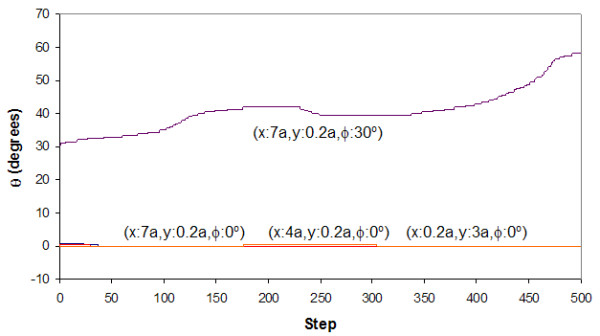
**The trace of θ**. Trace of θ in relation to step trajectories when the top flake was released at selected x-y distances away from the initial position and rotated by an angle *φ *to the *x*-axis.

The landscape maps of Figures 4 to 7 provide us with the ability to analyze the trajectories of certain selected starting conditions. If we consider the case in which the top flake was moved initially to (*x *= 0.7*a, y *= 0.*2a, φ *= 0^°^), we see from Figures 8 and 9 that it took slightly more than 50 steps before the flake became immobile over a short distance (about 0.2*a*). From Figure 10, the flake moved towards the x-axis (resulting in *α *= 0^°^) when this occurred. Figure 11 informs us that the flake rotated when it encountered the local minima and then restored to its original orientation when it finally came to rest. The situation when the top flake was moved initially to (*x *= 0.4*a, y *= 0.2*a, φ *= 0^°^) is slightly different. While the flake encountered a local minimum to reside in, this happened at a longer distance away (about 3*a*) from Figures 8 and 9. The trajectories for *α *(Figure 10) and θ (Figure 11) were nonetheless unchanged. A significantly larger behavior change occurred when the top flake was moved initially to (*x *= 7*a, y *= 0.2*a, φ *= 30^°^). With this, the movement trajectory for AC and BC were gradual before the flake eventually came to a rest. The interesting behavior is denoted in Figure 11 wherein the flake is seen to rotate in order to overcome the potential barriers in the way of motion. This is the basis of lubricity as noted previously [17]. When the top flake was moved initially to (*x *= 0.2*a, y *= 3*a, φ *= 0^°^), a significant degree of self-retraction was achieved (Figures 8 and 9). It is noted that the flake moved slightly towards the right (see Figure 10) in the process but exhibited little rotation (Figure 11).

We highlight certain caveats to the results provided here. The position and orientation tracing approach here assumes that there is no defect in either of the interacting flakes. The ability for graphite layers to be deform has been studied [23], and it is possible that such defects may strongly affect the self-retraction behavior [24]. This will be more evident for micron-scale flakes where the creation of entire interacting regions with perfect lattices is understandably more difficult. Such lattices should, however, be more achievable if the dimensions of the flakes are smaller. It is also important to note that the model here assumes that the force to move the top flake does not comprise an axial component that deforms the lattice. In previous demonstrations of self-retraction behavior, a mechanical probe that depressed and pulled the top flake laterally was utilized [16,24] in which such an effect cannot be ruled out. The measure of moving the flake with minimal axial deformation will most likely be accomplished using either optical [25], magnetic [26], electrical quadruple ions [27], or vibration [28] that have recently been reported with graphene sheets. The vibration of graphene nanostrips, it appears, offers the possibility of creating bending resonators with high sensitivity to environmental change [28]. It is conceivable, based on the findings here, for metastable misaligned states to appear with each cycle of actuation, to the extent of leaving a wrinkling effect on the sheets [29]. It is also noteworthy that long strips can result in gravity causing the structure to flop downwards. Remedy, however, is available by adopting a dangling arrangement, which has been demonstrated workable in applications associated with wetting monitoring [30,31].

## Conclusions

In summary, we have investigated the self-retraction of a graphite flake over another flake of the same size. This was done using a registry dependent interlayer interaction potential previously reported that is sensitive enough to account for the registry of the honeycomb structures and its layers. Under the assumption that the mass of the flake is small, the retraction process can be depicted as a series of quasi-static steps that seek the lowest potential. The results show that while there is an overall impetus to retract to the original position and orientation to restore to the lowest potential, there is a possibility for the return trajectory to encounter local potential minima that prevents the top flake from restoring fully. Essentially, this means that the graphite flake is able to assume meta-stable states.

Such a behavior can have significant impact in the creation of ordered graphene stacks for various applications. The maps of the parameters that we interrogated here provide the ability for precautions to be undertaken. For instance, any movement in the y direction keeping × and *φ *constrained will generally result in total retraction. Alternatively, it might be desirable to attain the meta-stable positions and orientations in order to obtain a different photonic or electrical behavior from an aligned stack. The maps then provide a road map to accomplish this. Such a behavior allows one to contemplate the creation of an array of microflake stacks in which the metastable states permit different information to be encoded by virtue of the differentiated photonic or electrical characteristics readable from each array site. Using the maps, these metastable states may be altered to or 'erased' using the retraction behavior to the original stable state. This portends the possibility of high density data recording and retrieval at the nanometer scale. The understanding of the metastable states may also shed light into the wrinkling behavior of graphene sheets, particularly if strips of them are to be used as resonators.

## Competing interests

The authors declare that they have no competing interests.

## Authors' contributions

TWN directed and provided the critical analysis of the work, CYL, EBC, and NT performed the simulations and refined various implementations of them. JLZ provided critique to the analysis results and developed some numerical checking simulations. JS provided logistical critique to the work. All authors read and approved the final manuscript.
